# Long non-coding RNAs in the alkaline stress response in sugar beet (*Beta vulgaris* L*.*)

**DOI:** 10.1186/s12870-020-02437-w

**Published:** 2020-05-20

**Authors:** Chunlei Zou, Yubo Wang, Bin Wang, Dan Liu, Lei Liu, Zhijia Gai, Caifeng Li

**Affiliations:** 1 College of Agronomy, Northeast Agricultural University, Harbin, 150030 People’s Republic of China; 2 Jiamusi Branch, Heilongjiang Academy of Agricultural Sciences, Jiamusi, 154000 People’s Republic of China

**Keywords:** Long noncoding RNAs, *Beta vulgaris* L., Alkaline stress, RNA sequencing, Computational analysis, Functional annotation

## Abstract

**Background:**

Long noncoding RNAs (lncRNAs) play crucial roles in regulating numerous biological processes in which complicated mechanisms are involved. Nonetheless, little is known about the number, features, sequences, and possible effects of lncRNAs on plant responses to alkaline stress.

**Results:**

Leaf samples collected based on the control *Beta vulgaris* L., as well as those under short-term and long-term alkaline treatments, were subjected to high-throughput RNA sequencing, through which a total of 8535 lncRNAs with reliable expression were detected. Of these lncRNAs, 102 and 49 lncRNA expression profiles were altered after short- and long-term alkaline stress, respectively. Moreover, 7 lncRNAs were recognized as precursors to 17 previously identified miRNAs. Four lncRNAs responsive to alkaline stress were estimated as targets for 8 miRNAs. Moreover, computational analysis predicted 4318 potential target genes as lncRNAs responsive to alkaline stress. Analysis of functional annotations showed that the abovementioned possible target genes were involved in various bioprocesses, such as kinase activity, structural constituents of ribosomes, the ribonucleoprotein complex and protein metabolic processes. Association analysis provided convincing proof of the interplay of specific candidate target genes with lncRNAs.

**Conclusion:**

LncRNAs likely exert vital roles during the regulation of the alkaline stress response and adaptation in plants through interaction with protein-coding genes. The findings of this study contribute to comprehensively examining lncRNAs in *Beta vulgaris* L. and shed more light on the possible roles and modulating interplays of lncRNAs responsive to alkaline stress, thereby laying a certain basis for functional analyses of these types of *Beta vulgaris* L. lncRNAs in the future.

## Background

Salt stress greatly affects the growth and development of plants through osmotic stress and ion imbalance [1]. Because of the large areas of distribution of saline soil and its negative affect on crop production, salt tolerance mechanisms of plants have been explored in depth over the last century [2]. Previous relevant studies have mainly focused on salt resistance mechanisms under neutral salt stress. Many fewer studies have been conducted on the responses of plants to stress from alkaline salts. Alkaline salt stress (NaHCO_3_ and Na_2_CO_3_), which is called alkaline stress for short, inhibits plant growth and survival more severely than neutral salt [3]. There are numerous common factors between neutral salt stress and alkaline stress, such as ion toxicity as well as osmotic stress [4]. Nonetheless, alkaline stress displays its uniqueness since the high pH value initiates malondialdehyde (MDA) and reactive oxygen species (ROS) production, thereby damaging the intracellular components and cell membrane in plants. As a result, alkaline stress represents a different stress form, and a quite complicated mechanism is involved in the alkaline tolerance of plants. The mechanism of tolerance to saline-alkaline stress in plants involves various gene expression profiles and gene product interactions but not single gene expression [5, 6].

Based on the improved high-throughput sequencing technique, more than 90% of the genome is suggested to produce numerous noncoding RNAs (ncRNAs) [7, 8]. When divided based on length, ncRNAs can be divided into long noncoding RNAs (lncRNAs), small interfering RNAs (siRNAs), and small RNAs such as microRNAs (miRNAs) [9, 10]. Among them, lncRNAs, which are over 200 nucleotides in length, have a low ability to code proteins, and they occupy the vast majority of ncRNAs [11, 12]. The expression profiles of lncRNAs are frequently cell- or tissue-specific, with their transcripts being located in the subcellular compartments [13]. Furthermore, in accordance with the positions with respect to the genomic protein-encoding genes, lncRNAs are divided into anti-sense, sense, bidirectional, intergenic, and intronic categories [14].

There is plenty of evidence that strongly proves that lncRNAs exert vital modulating parts within several plant bioprocesses [12, 15]. In addition, several lncRNAs have been identified to modulate gene expression in the close (*cis-acting*) or distant (*trans-acting*) genome through diverse mechanisms, such as promoter activity modification through repositioning of nucleosomes, DNA methylation, histone modification, accessory protein activation/gathering/transportation, repression, and epigenetic silencing [15, 16]. More studies are carried out to reveal the lncRNA functions in plants. For instance, *At4* and *AtIPS1* are identified to be *miR399-*specific mimics through sequestering and binding *miR399*, as well as reducing *PHO2* cleavage mediated by *miR399*, which plays a vital role in phosphate uptake [17]. In addition, Swiezewski et al. [18] changed *FLOWERING LOCUS C* (*FLC*) expression in Arabidopsis and discovered that lncRNAs participate in regulating flowering. *LDMAR* in rice modulates sterility in males, which is sensitive to photoperiod [19]. Furthermore, *PINOID* represents an important factor for regulating the transport of polar auxin, and lncRNA *APOLO* expression can induce changes in the formation of chromatin to upregulate *PINOID* expression [20]. Altogether, 13,087 and 11,641 lncRNAs in *M. truncatula* were identified to display responses to salt and osmotic stresses, respectively; meanwhile, 5634 lncRNAs have been suggested to exhibit responses to both salt and osmotic stresses [21]. In Arabidopsis, lncRNA *HID1* is found to mediate red light-induced photomorphogenesis [22]. *PDIL1* inhibits *MtPHO2* downregulation, and the latter encodes the miR399-regulated ubiquitin-conjugating E2 enzyme, whereas *PDIL2* and *PDIL3* show direct transcriptional regulation of phosphate transport. Numerous studies have made tremendous progress, but in comparison with mammalian lncRNAs, plant lncRNA functions, together with the related regulatory networks, remain largely unclear. lncRNAs are systemically identified in a small portion of plants, such as Arabidopsis [23, 24], rice [25], maize [26], poplar [27] and *Medicago truncatula* [28]. However, the roles of lncRNAs within the Chenopodiaceae sugar beet (*Beta vulgaris* L*.*) model remain largely unknown.

Sugar beet (*Beta vulgaris* L*.*), a critical economic crop, makes a great contribution to sugar supply globally. It is not only used in the food industry but also used as a renewable energy source [29]. Sugar beets can adapt to both abiotic and biotic stresses, including salinity, drought, heat, and cold, under a temperate climate [30]. Sugar beet, a crop that exhibits high tolerance to salt, has been adopted for investigating crop adaptation to sodium chloride (NaCl) as a good model. Substantial previous studies on the response to saline stress have been conducted at physiological and molecular levels, such as antioxidant enzymes, proteomes and transcriptomes [31–33]. Sugar beet genome sequencing has been completed [34]; as a result, sugar beet has been utilized as a superb model to investigate stress tolerance and response in plants. However, lncRNAs involved in the sugar beet response to alkaline stress have never been reported.

In this study, the lncRNAs responsive to alkaline stress that were detected within sugar beet leaves were detected and characterized comprehensively at a genome-wide level; in addition, the candidate genes and miRNAs interacting with the identified lncRNAs were predicted. Overall, the results shed more light on the *Beta vulgaris* L*.* lncRNAs that were responsive to alkaline stress and laid the foundation to investigate the as-identified lncRNA functions.

## Results

### Alkaline stress impacted physiological and growth features

**Fig. 1:**
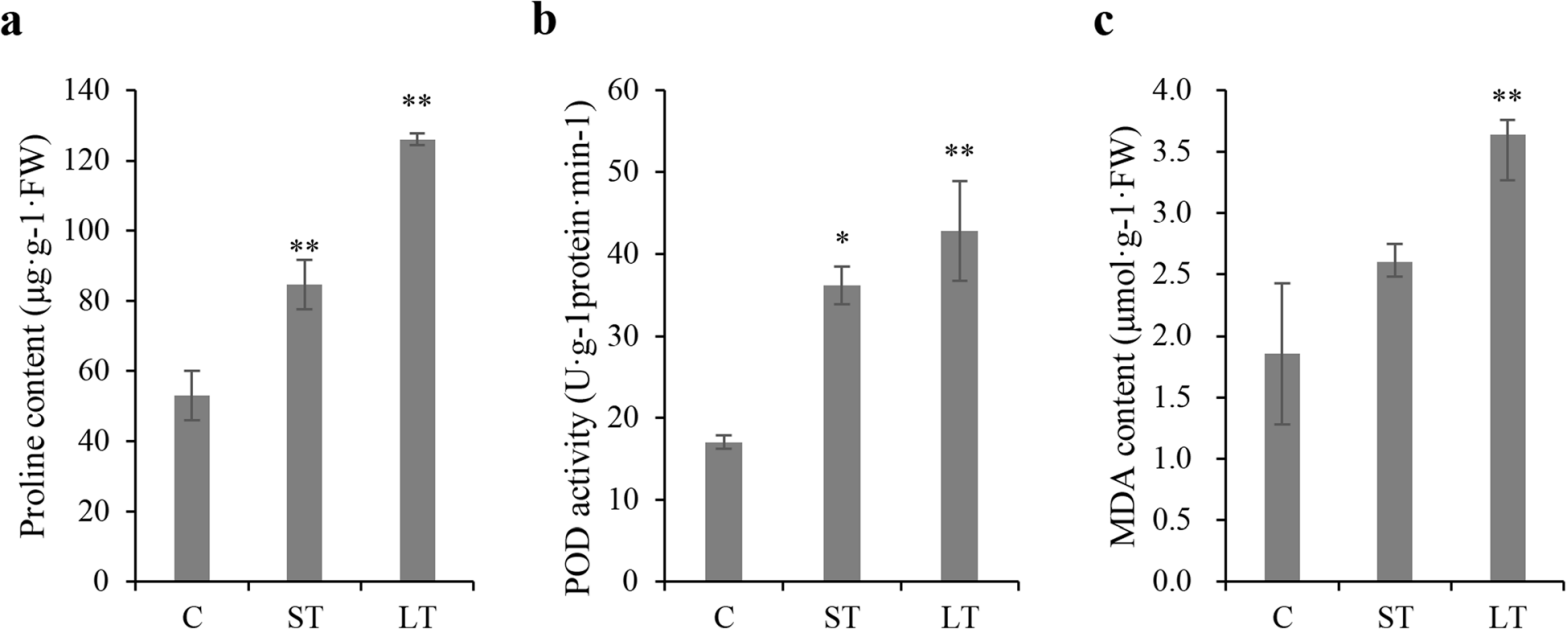
Effects of alkaline treatment on physiological characteristics. **a**-**c** Changes in proline concentration, malondialdehyde (MDA) level, and peroxidase (POD) activity in leaf samples harvested from 0-day, 3-day, and 7-day treated plants. C represents control samples, ST represents 3-day alkaline treated samples, and LT represents 7-day alkaline challenged leaves. The error bar suggests SDs across 2 different biological duplicates (*n* = 3). Asterisks represent significant differences in alkaline-challenged leaves compared with controls (*P < 0.05; **P < 0.01)

To examine the effects of alkaline treatments with different lengths of time on the physiological characteristics of sugar beet, we measured the proline concentration, MDA content, and POD activity within leaf samples collected from 0-day, 3-day, and 7-day alkaline-treated plants. As shown in Fig. 1a-c, alkaline treatments with different lengths of time significantly affected these three physiological characteristics. The abovementioned heterogeneities in physiology suggested the presence of obvious alterations within gene expression (including lncRNAs) of sugar beet after alkaline treatment.

To examine the effects of long-term alkaline treatment on sugar beet, we measured the growth and photosynthetic characteristics of plants in hydroponics with or without alkaline treatment for 7 days. Morphological changes were observed after alkaline treatment (Additional file Fig. S1a). Alkaline treatment significantly inhibited plant growth. The photosynthetic characteristics showed obvious changes following alkaline stress (Additional file Fig. S1b-d). For instance, T_r_, G_s_ and P_n_ of alkaline-treated plants remarkably decreased compared with the controls (Additional file Fig. S1b-d). However, there was no significant difference in photosystem II (Y (II)) quantum yield between the control and alkaline-challenged plants. The significant changes in photosynthetic and growth characteristics indicated the inhibition of alkalinity in plant growth.

### Sugar beet lncRNA characterizations

**Fig. 2:**
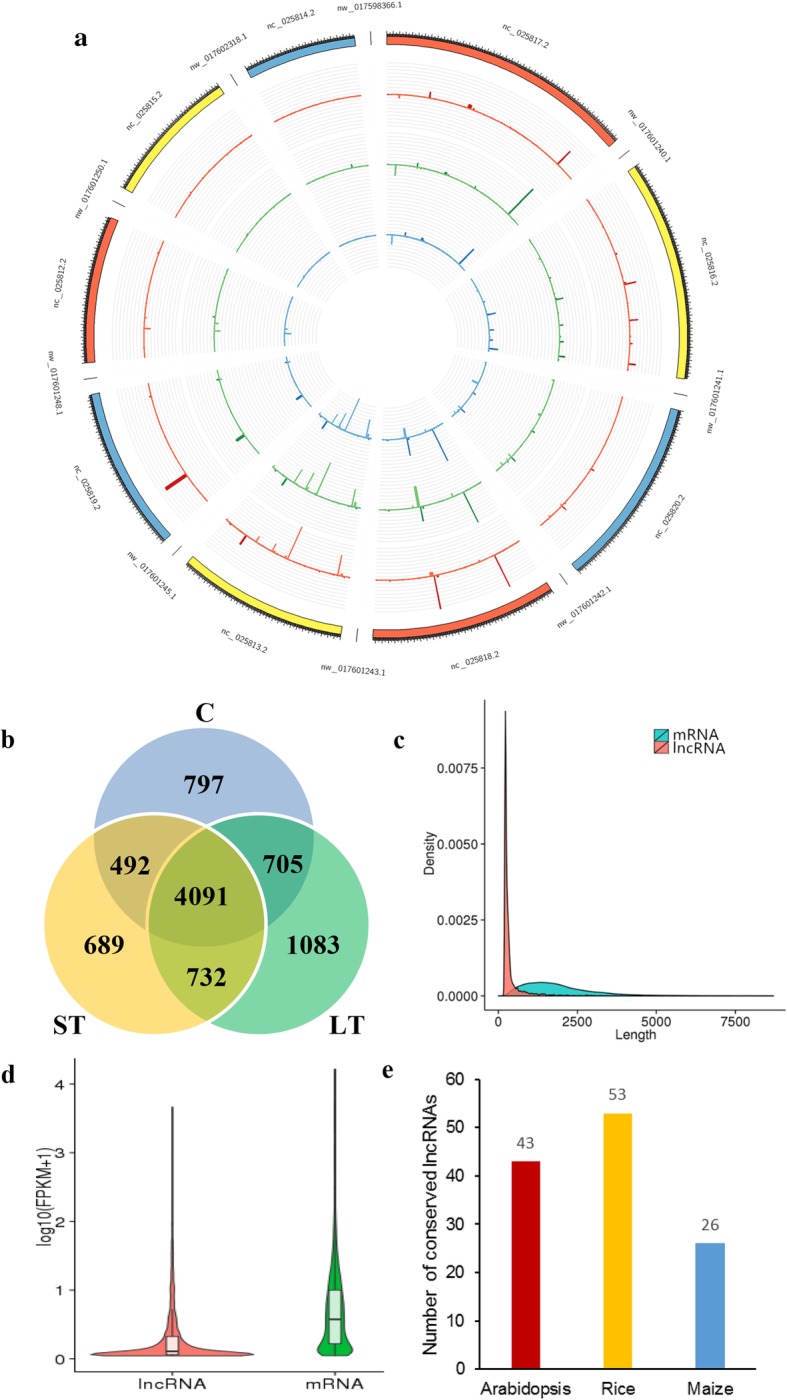
Sugar beet lncRNA features. **a** LncRNA distribution in every chromosome. It comprises three concentric rings, including C, ST and LT successively outside in. C represents control samples, ST represents short-term alkaline treated samples, and LT represents long-term alkaline treated samples. **b** Venn diagram for the specific and common lncRNAs among C, ST and LT leaves. **c** lncRNAs have a short length relative to the protein-encoding transcripts. **d** lncRNA expression is markedly downregulated compared with mRNA expression. **e** LncRNA conservation. The number of *P. tomentosa* lncRNAs was conserved within the genomes of *Zea mays*, *Oryza sativa*, and *Arabidopsis thaliana*. These conserved lncRNAs were deemed lncRNAs having > 20% matched sequences to other genomes

According to the abovementioned alterations in physiological features, leaf samples were collected from control, 3-day alkaline and 7-day alkaline-challenged plants to carry out high-throughput RNA sequencing. Afterwards, lncRNAs were identified systemically in the whole genome, which identified 6085, 6004 and 6611 lncRNAs (FPKM> 0.5) based on the control, short-term and long-term alkaline-challenged plant libraries, respectively (Fig. 2b). A total of 8535 lncRNAs with reliable expression (FPKM> 0.5 for 1 or over 1 library) were identified, including 2051 antisense and 6034 sense lncRNAs.

Furthermore, 8535 lncRNAs were characterized for their basic genomic characteristics. The lncRNA distribution within *Beta vulgaris* L*.* chromosomes was examined, which indicated the average density of lncRNAs of 15.06 lncRNAs for every Mb (Fig. 2a). The lncRNA transcript lengths followed the range of 201–12,882 (average, 424) nucleotides, and they were short compared with those of *Beta vulgaris* L*.* protein-encoding genes (average, 1998 nucleotides) (Fig. 2c). lncRNA expression patterns had low mean counts (FPKM = 15.83) relative to those in coding transcripts (FPKM = 19.11) (Fig. 2d). Based on the results of conservation analysis, only a few *Beta vulgaris* L. lncRNAs exhibited conservation within maize, rice and Arabidopsis (Fig. 2e). In addition, according to BLAST analyses on the lncRNAs of *Beta vulgaris* L. relative to the ncRNAs NONCODE database, 98.6% of our detected lncRNAs were specific in *Beta vulgaris* L.

Moreover, the sequences of lncRNAs were investigated to determine their potential as targets or precursors for the previously identified miRNAs. The miRNA precursors were aligned to 8535 lncRNAs, and three lncRNAs were suggested to be the precursors of two already identified miRNAs (Table 1). For instance, the lncRNA *LNC_003048* was estimated to be the *gma-miR4995* precursor.

**Table 1:** LncRNAs corresponding to miRNA precursors

lncRNA ID	Class	miRNA ID	Mature sequence of miRNA
*LNC_003498*	Sense	*gma-miR4995*	AGGCAGUGGCUUGGUUAAGGG
*LNC_003048*	Sense	*gma-miR4995*	AGGCAGUGGCUUGGUUAAGGG
*LNC_003418*	Sense	*hbr-miR6173*	AGCCGUAAACGAUGGAUACU

### Identification of alkaline-responsive lncRNAs

**Fig. 3:**
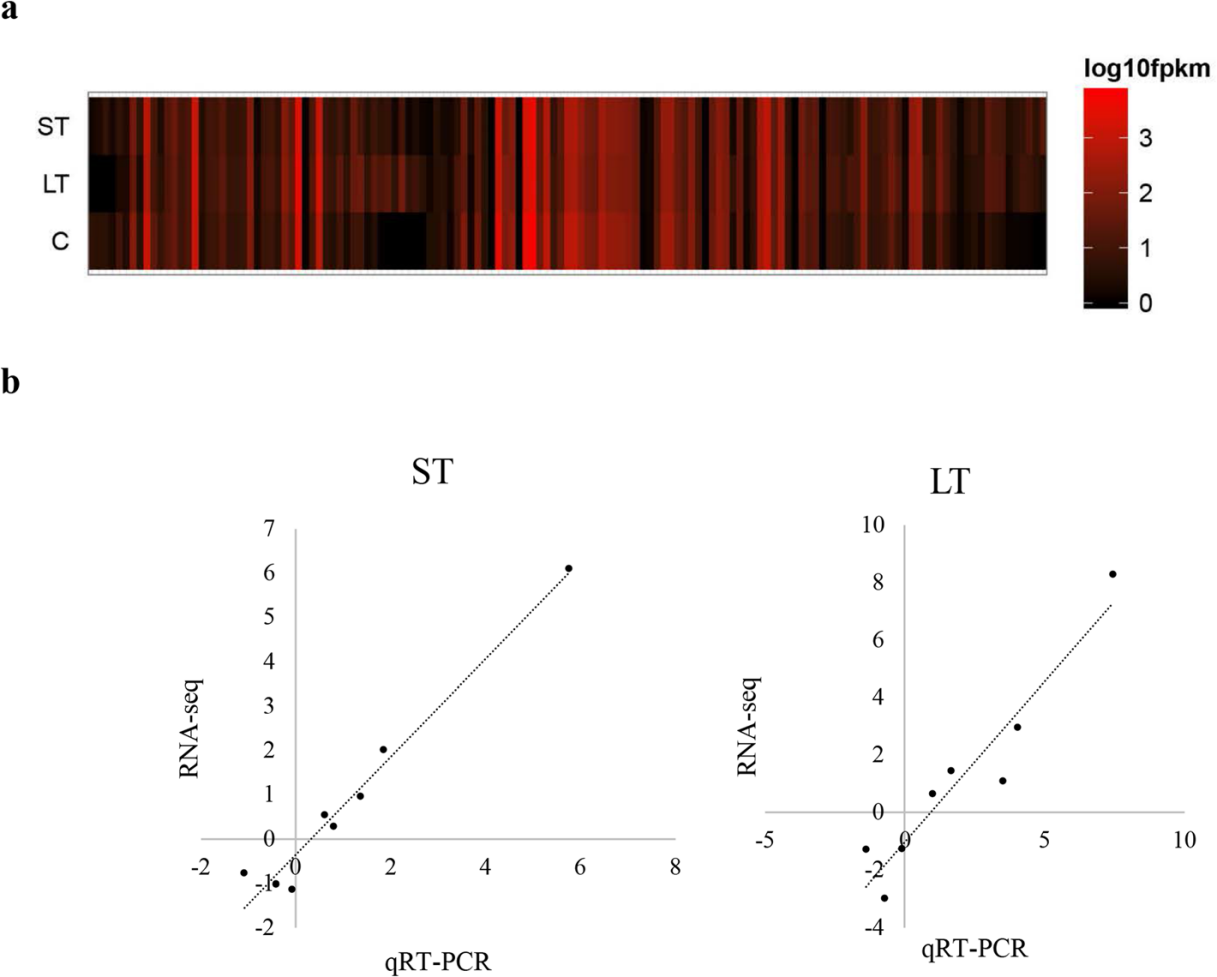
Alkaline-responsive lncRNA expression. **a** Heat map representing the expression levels of alkaline-responsive lncRNAs in response to short- and long-term stress. **b** RNA-seq data validated through qRT-PCR within samples under short- and long-term alkaline stress

We identified 93 lncRNAs (Additional file Table S1) with alkaline-responsive expression patterns (*P* < 0.05), with 24 upregulated and 40 downregulated under short-term alkaline treatment and 26 upregulated and 11 downregulated under long-term alkaline treatment (Fig. 3a). Of these alkaline-responsive lncRNAs, five lncRNAs were upregulated by both short-term and long-term alkaline stresses, whereas three lncRNAs were downregulated by both stresses. Among those differentially expressed lncRNAs, 10 and 2 had an increase of > 4-fold as well as a decrease of > 4-fold in response to short-term alkaline stress, respectively, and 9 and 4 showed a 4-fold increase and a > 4-fold decrease in response to alkaline challenge for a long term, respectively.

To confirm the expression response to alkaline stress, 8 lncRNAs were screened, and the specific expression profiles were verified through qRT-PCR. According to Table 2, the RNA-seq and qRT-PCR analyses revealed identical variations in expression profiles, regardless of certain heterogeneities in expression. For instance, qRT-PCR and RNA-seq analyses revealed *LNC_008366* upregulation in response to long- and short-term alkaline treatments. Plotting for qRT-PCR and RNA-seq data (Fig. 3b) revealed a great coefficient of correlation (R^2^ = 0.964, *P* < 0.05 for short-term alkaline challenged samples and R^2^ = 0.902, *P* < 0.05 in long-term alkaline treated samples) between the two datasets.

**Table 2:** LncRNAs used to validate RNA-seq data through qRT-PCR

LncRNA ID	RNA-seq		qRT-PCR	
	Short-term	Long-term	Short-term	Long-term
*LNC_001194*	2.02	2.97	1.86	4.04
*LNC_007400*	0.96	1.46	1.38	1.67
*LNC_008363*	0.54	1.10	0.62	3.51
*LNC_008366*	6.1	8.29	5.76	7.45
*LNC_008534*	0.28	0.66	0.81	1.00
*LNC_000365*	−0.77	−1.29	−1.08	−1.37
*LNC_004675*	−1.02	−2.98	−0.4	− 0.71
*LNC_007731*	−1.13	−1.26	−0.07	−0.11

### Candidate target genes for lncRNAs responsive to alkaline stress and their functions

LncRNAs play vital roles in the regulation of gene expression profiles; therefore, it may be helpful to identify and analyze specific target genes to examine their related functions. According to computational prediction, a total of 133 candidate target genes were screened for lncRNAs responsive to alkaline stress (Additional file Table S2).

**Fig. 4:**
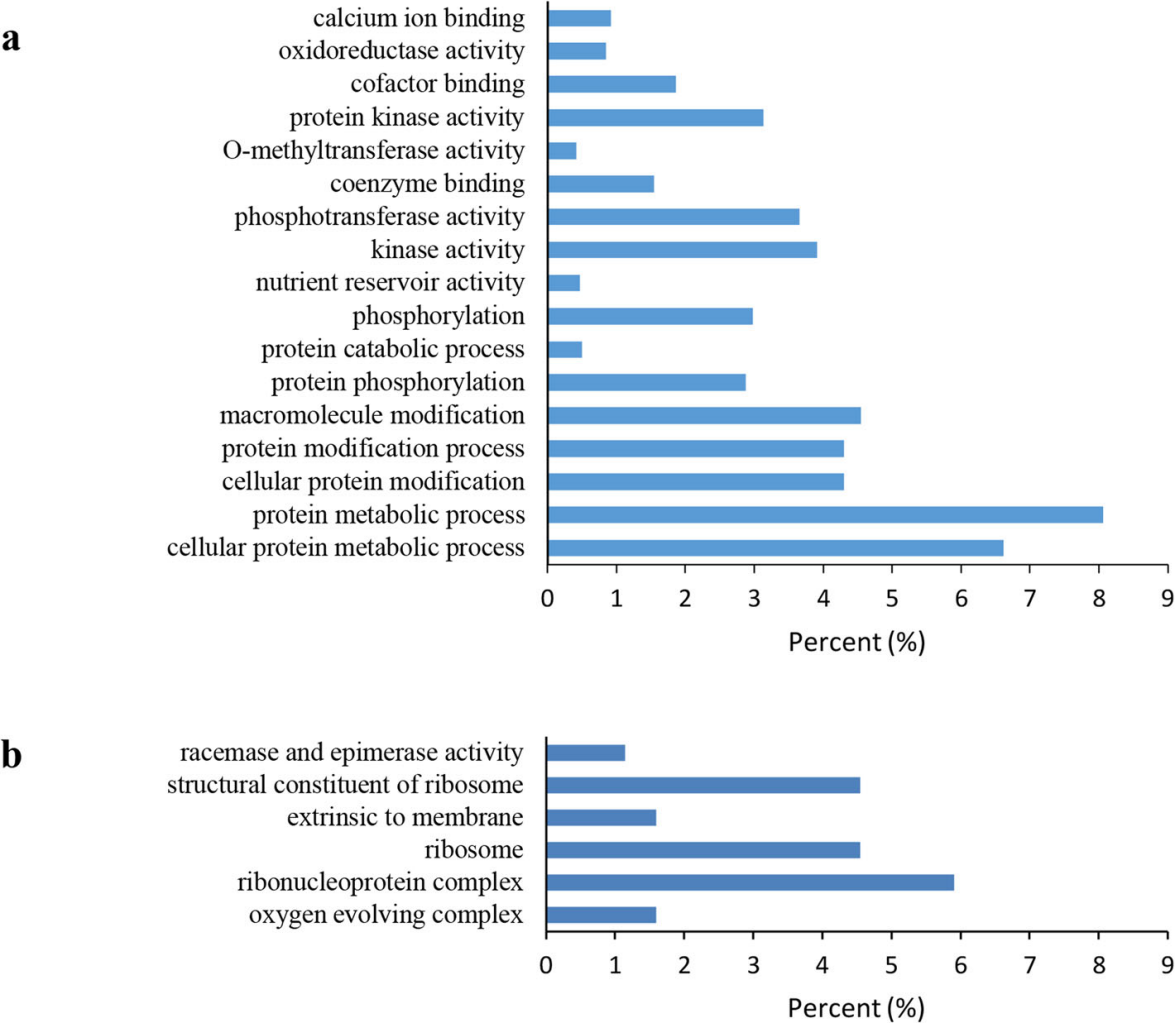
Gene ontology (GO) for potential genes of alkaline-responsive lncRNAs (**a**) GO analysis at short-term alkaline treatment (**b**) GO analysis for long-term alkaline treatment

As suggested in prior works, lncRNAs are more likely to be located close to their regulated genes [35–38]. To reveal the possible effects of those as-identified lncRNAs, Gene Ontology (GO) analysis was conducted on alkaline-responsive lncRNA-targeting genes. Six and 17 GO terms were markedly enriched (*P* < 0.05) in samples under long- and short-term alkaline challenge, respectively (Fig. 4). The major category of molecular functions was kinase activity (GO:0016301) as well as ribosomal structural constituent (GO:0003735). Genes involved in the ribonucleoprotein complex (GO:0030529) showed high representativeness of cell components. With regard to the bioprocess, the metabolic process of proteins (GO:0019538) was the most representative GO term, while the metabolic process of cellular protein (GO:0044267) ranked second. Protein metabolic process showed high representativeness in each GO term, which involved 482 genes. The above results indicated that the lncRNAs responsive to alkaline might modulate genes participating in numerous bioprocesses, such as energy synthesis, signal transduction, detoxification, molecule metabolism, translation and transcription in response to slat and osmotic stresses.

Under stress, numerous GO terms showed significant enrichment, including the metabolic process of protein (GO:0019538) as well as the ribonucleoprotein complex (GO:0030529), which showed high significance in leaves under short-term and long-term alkaline stresses (Fig. 4). The 2-methyl-6-phytyl-1 gene *LOC104894575*, which belonged to the abovementioned 2 GO terms, increased under short-term alkaline stress. *LOC104894575* was estimated to be under the regulation of lncRNA *LNC_000365*, coexpressing with *LOC104894575*. Protein metabolism is an important biological process in plants to regulate growth and development and cope with environmental stress [39]. Our results suggested that *LNC_000365* may regulate protein metabolism by modulating *LOC104894575* expression.

Under abiotic stress conditions, signal transduction networks will be motivated to manage such stressful environments. It has been suggested that the phospholipid metabolic pathway plays a vital role in responding to various abiotic challenges [40]. In the present study, the hypothetical protein isoform B gene *LOC104888232*, which belongs to GO:0046488 (phosphatidylinositol metabolic process), was downregulated under long-term alkaline stress, and the lncRNA *LNC_001194* was expressed 37.9 kb upstream of the *LOC104888232* coding sequence. According to the findings, *LNC_001194* might modulate *LOC104888232* expression.

Plants that are subjected to abiotic challenges may exhibit oxidative damage, which is evidenced by massive reactive oxygen species (ROS) accumulation, thereby damaging the membrane system. In response to excess ROS accumulation, plants arouse protective enzymes for eliminating ROS [41]. In this study, the expression of the gene *LOC104906740*, which encodes peroxidase (POD), increased in response to alkaline treatment in the short term. We identified the lncRNA *LNC_007731* coexpressing with *LOC104906740*. These results suggested that *LNC_007731* might participate in the regulation of OS tolerance in plants by regulating POD expression.

The effects of alkalinity on inhibiting the growth of plants are classified as pH stress, osmotic stress and ionic toxicity [42]. Plants frequently show similar tolerance mechanisms, including the changed transduction of phospholipid signals, energy synthesis, and detoxification to saline or alkaline stress [43]. In the present study, the ion transporter genes *LOC104906281* and *LOC104892091* were both upregulated by short-term alkaline stress. We identified the lncRNA *LNC_000365* coexpressing *LOC104892091*. These results suggested that *LNC_000365* may be involved in ion compartmentation. The peptidyl-proline modification gene *LOC104888024* and betaine aldehyde dehydrogenase gene *LOC104894203* were upregulated by long-term and short-term alkaline stress, respectively. The lncRNA *LNC_004748* was predicted to be coexpressed with *LOC104888024* and *LOC104894203*. These results suggested that *LNC_004748* may be involved in osmotic adjustment.

### Association of alkaline-responsive lncRNA expression with the related candidate target genes

**Fig. 5:**
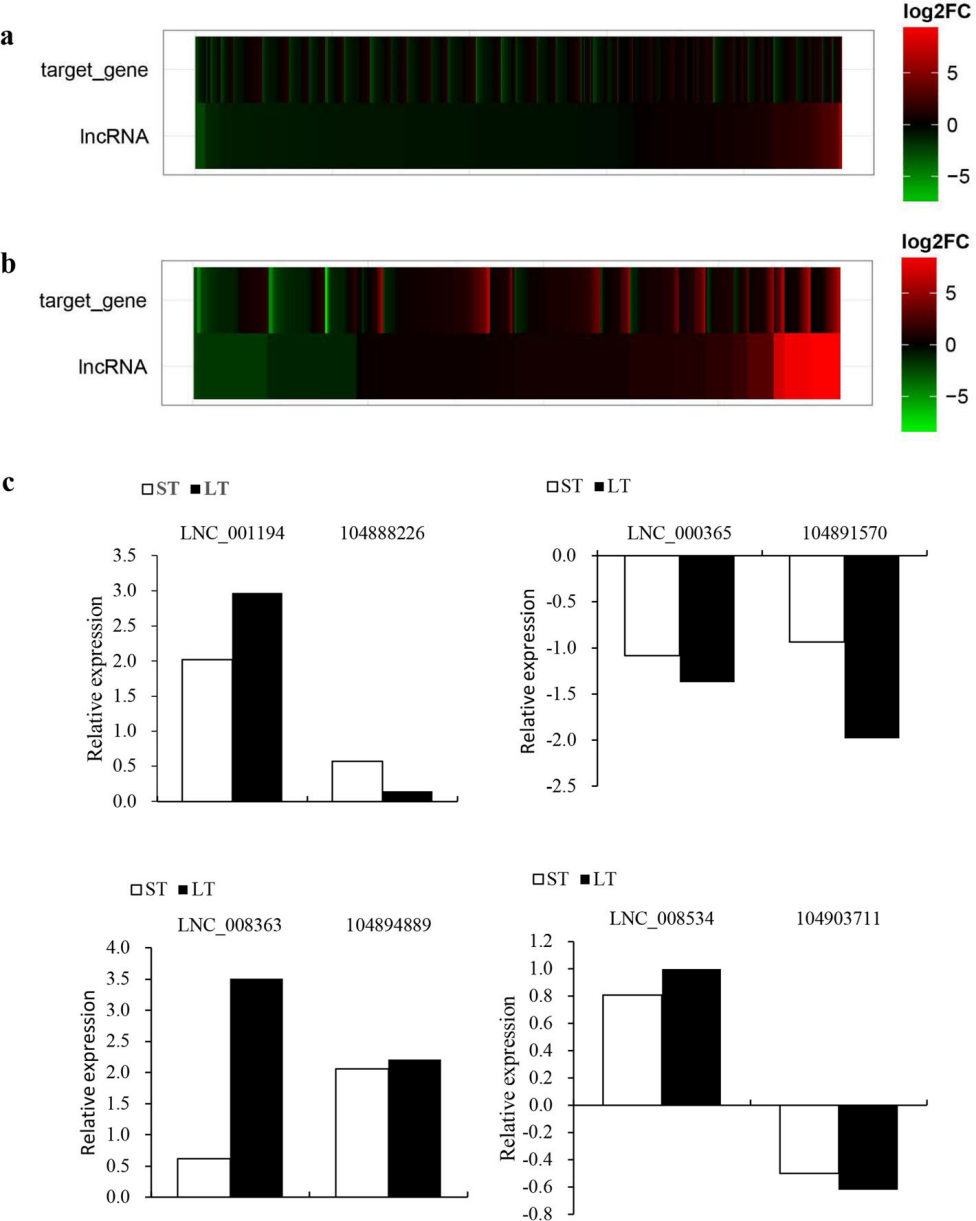
Relationship in the expression of alkaline-responsive lncRNAs with the corresponding candidate target genes. **a** Heat map suggesting alkaline-responsive lncRNAs, together with corresponding candidate target gene expression under short-term stress. **b** Heat map representing alkaline-responsive lncRNAs, as well as the corresponding candidate target genes under long-term stress. **c** Association of alkaline-responsive lncRNAs with corresponding candidate target genes validated by qRT-PCR. ST represents short-term saline-alkaline stress samples, and LT represents long-term saline-alkaline stress samples. The error bar indicates SDs across 3 biological duplicates (*n* = 3)

Following the prediction of candidate target genes for those lncRNAs responsive to alkaline stress, the changes in candidate target gene expression were determined following alkaline stress. Of those 133 candidate colocation target genes, 4 and 4 exhibited significant variations in response to short-term and long-term alkaline stress at the transcript level (*P* < 0.05); with regard to candidate coexpression target genes, 516 and 210 out of 3492 displayed significant variations at the transcript level (*P* < 0.05). The abovementioned candidate target genes showing alkaline-responsive (*P* < 0.05) expression levels might serve as alkaline-responsive lncRNA targets. To analyze the association of alkaline-responsive lncRNA expression with the candidate target genes showing alkaline-responsive (*P* < 0.05) expression profiles, the expression trends following alkaline stress were compared. The expression levels for 1550 (65.8%) and 807 (34.2%) lncRNA–gene pairs (65.8%) showed the same and opposite trends, respectively, in response to short-term alkaline treatment (Fig. 5a), whereas 243 (65.9%) and 126 (34.1%) lncRNA-gene pairs showed the same and opposite expression trends, respectively, in response to long-term alkaline treatment (Fig. 5b). Therefore, a majority of those target genes with differential expression displayed an identical trend to related lncRNAs following alkaline stress. Moreover, 4 pairs of lncRNAs and target genes were screened, and the specific expression levels were examined through qRT-PCR. The associations of lncRNA expression with candidate target genes recognized through qRT-PCR were the same as those recognized through RNA-seq (Fig. 5c). For instance, *LNC_008363* together with its potential target gene (*LOC104894889*) was upregulated in response to long- and short-term alkaline treatment, which shared an identical trend of expression. In addition, the heterogeneous relationships in the expression of alkaline-responsive lncRNAs compared with the specific candidate target genes revealed the diverse lncRNA regulating mechanisms.

### Alkaline-responsive lncRNAs related to photosynthetic carbon assimilation

Carbon assimilation plays a pivotal role in plant growth and development. In the present study, many target genes of alkali-responsive lncRNAs were related to photosynthetic carbon assimilation (Table 3). For instance, the expression of the photosystem I subunit O gene *LOC104907718* and glucose-6-phosphate 1-dehydrogenase gene *LOC104897526* were regulated by lncRNA *LNC_004949*, which was downregulated more than twofold under short-term alkaline treatment. Three target genes of *LNC_007731* (downregulated more than twofold under long-term alkaline treatment) were carbonic anhydrase gene *LOC104902037*, magnesium-protoporphyrin IX monomethyl ester [oxidative] cyclase gene *LOC104889639* and glucose-6-phosphate 1-dehydrogenase gene *LOC104897526*. Overall, the results showed that lncRNAs may participate in photosynthetic carbon fixation by regulating the expression of related genes.

**Table 3:** Some target genes of alkaline-responsive lncRNAs related to photosynthetic carbon assimilation

Comparison group	lncRNA ID	*P*-value	Target gene id	Target gene description
ST_vs_C	*LNC_004949*	−1.396038917	*LOC104907718*	photosystem I subunit O
	*LNC_004949*	−1.396038917	*LOC104897526*	glucose-6-phosphate 1-dehydrogenase
	*LNC_007731*	−1.126389158	*LOC104897387*	carbonic anhydrase 2
	*LNC_007731*	−1.126389158	*LOC104893033*	glycerol-3-phosphate acyltransferase
	*LNC_007731*	−1.126389158	*LOC104902037*	carbonic anhydrase
	*LNC_007731*	−1.126389158	*LOC104900039*	photosystem II repair protein PSB27-H1
	*LNC_007731*	−1.126389158	*LOC104897526*	glucose-6-phosphate 1-dehydrogenase
LT_vs_C	*LNC_000365*	−1.291328691	*LOC104902037*	carbonic anhydrase
	*LNC_000365*	−1.291328691	*LOC104908971*	magnesium protoporphyrin IX methyltransferase
	*LNC_000365*	−1.291328691	*LOC104890676*	chloroplast stem-loop binding protein of 41 kDa b
	*LNC_000365*	−1.291328691	*LOC104889354*	magnesium-chelatase subunit ChlH
	*LNC_000365*	−1.291328691	*LOC104900584*	magnesium-chelatase subunit ChlI
	*LNC_000365*	−1.291328691	*LOC104889639*	magnesium-protoporphyrin IX monomethyl ester [oxidative] cyclase
	*LNC_007731*	−1.261549284	*LOC104902037*	carbonic anhydrase
	*LNC_007731*	−1.261549284	*LOC104889639*	magnesium-protoporphyrin IX monomethyl ester [oxidative] cyclase
	*LNC_007731*	−1.261549284	*LOC104897526*	glucose-6-phosphate 1-dehydrogenase

## Discussion

For the time being, lncRNAs have been systemically identified only in several plants. The current work discovered 8535 lncRNAs with reliable expression and revealed the similar characteristics of sugar beet (*Beta vulgaris* L.) lncRNAs with those found in other species. First, based on prior works in zebrafish, humans, rice, cucumber, and Arabidopsis, compared with protein-encoding transcripts, lncRNAs have a short length with markedly reduced expression [13, 25, 38, 44, 45]. Second, unlike miRNAs that show high conservation across various plant species [46], lncRNAs in plants display evolutionary constraints to a low extent. According to our BLAST analyses relative to sequences of maize, rice and Arabidopsis genomes, only a few *Beta vulgaris* L. lncRNAs were conserved (Fig. 2e). Additionally, BLAST analyses on lncRNAs of *Beta vulgaris* L. relative to the NONCODE database only identified the conservation of a few of our lncRNAs (1.4%). lncRNAs from the remaining plant species also display similar observations, including maize, rice Arabidopsis, cucumber, wheat, and Populus [25–27, 45, 47, 48]. Given such a low conservation level, such lncRNAs in plants can experience fast evolution. Third, analogous to lncRNAs from rice, Arabidopsis, humans, and other species [44, 49, 50], some lncRNAs in *Beta vulgaris* L. have been identified as target mimics or miRNA precursors (Table 1). The abovementioned findings suggest that the association of miRNAs with lncRNAs may play a vital role in lncRNAs. Thus, this study offered abundant evidence to investigate the functions of *Beta vulgaris* L. lncRNAs.

lncRNAs have been suggested in prior works to exert vital roles in various bioprocesses. Nonetheless, the alkaline-responsive lncRNA functions are not yet completely understood. lncRNAs are reported to exert their functions through interactions with miRNAs and target genes [51, 52]. As a result, it is an efficient approach to predict and analyze the miRNAs and target genes interacting with lncRNAs to examine specific lncRNA functions. The candidate target genes were predicted for alkaline-responsive lncRNAs in the current work. Furthermore, we identified seven lncRNAs to be the precursors of 17 already identified miRNAs (Table 1) and four alkaline-responsive lncRNAs to be the targets for eight miRNAs from four families (Table 2).

Various responsive mechanisms have evolved in plants to release damage resulting from abiotic stresses [53]. Many protein-encoding genes were found to exert important roles in regulating the response to abiotic stress in plants, including *SOS1* and *DREB1A/CBF3* [54–57]. In addition, lncRNAs have been identified as powerful approaches in plants for enhancing their abiotic stress tolerance [58]. Consequently, recognition, functional characterization and regulatory network construction of stress-responsive lncRNAs shed more light on environmental stress tolerance in plants. Plant lncRNAs responsive to abiotic/biotic stresses have been discovered in some recent works. Liu et al. [23] found 6484 lincRNAs, among which 1832 responded to salinity, cold, drought, and abscisic acid. Recently, 504 lncRNAs responsive to drought were found in poplar [49]. Wang et al. [21] identified 471 lncRNAs responsive to salt and osmotic stresses in both root and leaf samples. Wang et al. [28] discovered a total of 10,785 lncRNAs from *Medicago truncatula*, the legume model species, among which 224 and 358 were phosphate deficiency-responsive in roots and leaves, respectively. In the present work, all lncRNAs, such as the antisense and sense lncRNAs, were discovered by the use of the state-of-the-art sequencing technique (strand-specific sequencing) as well as analysis approaches (such as Cuffcompare analysis). Moreover, specific and common lncRNAs were discovered from short- and long-term alkaline-treated leaf samples to investigate possible sugar beet lncRNA functions under alkaline challenge. To our knowledge, the current work is the first to report the systemic retrieval, characterization and analysis of lncRNAs isolated from short-term and long-term alkaline-treated leaf samples of sugar beet using high-throughput sequencing.

In the present study, 17 and 6 GO terms were significantly enriched (*P* < 0.05) within samples subjected to short- and long-term alkaline stresses, respectively (Fig. 4). The major categories of molecular functions, cellular components and biological processes were kinase activity (GO:0016301), ribonucleoprotein complex (GO:0030529) and protein metabolic process (GO:0019538), respectively, which were similar to those of Wang et al. [21]. Moreover, in this study, several lncRNAs were estimated to modulate gene expression in response to abiotic stress. For example, the lncRNA *LNC_007731* coexpressing with the gene *LOC104906740*, coding for peroxidase, and peptidyl-proline modification gene *LOC104888024* and betaine aldehyde dehydrogenase gene *LOC104894203* were regulated by the lncRNA *LNC_004748*. The above findings demonstrated the vital parts of lncRNAs in response to environmental stress in plants.

In addition to the potential interaction with specific target genes, lncRNAs responsive to alkaline stress also show interactions with miRNAs. Three lncRNAs were discovered in the current work to be candidate targets for eight miRNAs from four families (Table 3). According to the findings, the lncRNAs responsive to alkaline stress might also impact numerous distinct bioprocesses through the interaction with miRNAs.

## Conclusions

A total of 8537 reliable lncRNAs were discovered from three *Beta vulgaris* L. RNA-seq libraries by means of high-throughput sequencing; among them, 102 and 49 responded to short- and long-term alkaline stresses, respectively. Of these alkaline-responsive lncRNAs, six lncRNAs were upregulated by both short-term and long-term alkaline stresses, whereas five lncRNAs were downregulated by both stresses. Among these lncRNAs responsive to alkaline stress, four were identified as targets for eight miRNAs from four families. After aligning the miRNA precursors to a total of 8535 lncRNAs, 7 lncRNAs were discovered to be the precursors for 17 already identified miRNAs. Moreover, the results of computational prediction revealed 4318 candidate target genes of lncRNAs responsive to alkaline stress. We found enrichments of GO terms in cellular structure, molecular function and biological process, including kinase activity, structural constituent of ribosome, ribonucleoprotein complex and protein metabolic process. An interaction network was established on the basis of genomic colocated and coexpressed protein-encoding genes and lncRNAs. According to our findings, lncRNAs may play a vital role in regulating plant responses as well as adaptation to alkaline treatment by interacting with protein-encoding genes. The abovementioned results shed valuable light on additional plant lncRNA functional characterizations in general as well as *Beta vulgaris* L. under abiotic stress.

## Methods

### Plant materials and treatment conditions

Pelleted sugar beet cultivar “KWS0143” seeds (supplied by KWS company, Germany) were germinated within vermiculite containing distilled water for a week. Then, the seedlings were irrigated with Hoagland solution (pH 6.85) for 4 weeks at 65% relative humidity, 25 °C/20 °C (day/night), light intensity of 450 μmol·m^− 2^·s^− 1^ and a light-dark cycle of 16 h–8 h dark. The components of the Hoagland solution are listed in Additional file Table S3. Finally, the seedlings were treated with a 75 mM alkaline solution mixture (NaHCO_3_:Na_2_CO_3_, 2:1, pH 9.67) for different periods of 0 days (control, designated as C), 3 days (short-term treatment, designated as ST) and 7 days (long-term treatment, designated as LT). Fresh leaves from the same position of seedlings in different treatments were collected, and 3 biological duplicates were prepared. Each collected leaf sample was frozen in liquid nitrogen immediately following sampling and preserved at − 80 °C for physiological parameter determination, RNA-seq and qRT-PCR assays.

### Measurement of physiological parameters

The activity of peroxidase (POD) was determined in accordance with Zhang’s protocol [59]. Typically, 1 unit POD activity was deemed to be the enzyme amount necessary for the catalysis of 1 μg substrate by 1 mg protein within 1 min at 37 °C. The malondialdehyde (MDA) content was measured as described by Li [60]. Plant tissues were collected into prechilled acetocaustin, followed by 10 min of centrifugation at 10,000 rpm. Thereafter, the supernatants were collected and blended with thiobarbituric acid, followed by transfer onto a boiling water bath for a period of 15 min. Following 10 min of centrifugation at 10,000 rpm, MDA content was measured at wavelengths of 600, 532 and 450 nm. The proline level was also detected in accordance with the ninhydrin colorimetric method [61].

The transpiration rate (Tr), stomatal conductance (Gs), and net photosynthetic rate (Pn) were determined based on three comprehensively extended leaves by the use of the CI-340 portable photosynthesis system (CID, Inc., USA). The Mini-PAM Fluorometer (Walz, Germany) was utilized to determine photosystem II (Y (II)) quantum yield under light conditions according to the description by Oelze et al. [62].

### Extraction of RNA, construction of library, and RNA-sequencing

Leaf samples from 0-day, 3-day and 7-day alkaline-treated plants (3 biological duplicates for each treatment) were utilized to carry out high-throughput RNA-sequencing (RNA-seq). Then, the Qiagen RNAeasy kit (Qiagen China, Shanghai, China) was used to extract total RNA in accordance with manufacturer protocols. The 1% agarose gels were utilized to monitor the contamination and degradation of RNA. A NanoPhotometer® spectrophotometer (IMPLEN, CA, USA) was employed to check the purity of the RNA. The Qubit® 2.0 A fluorometer equipped with the Qubit® RNA Assay Kit (Life Technologies, CA, USA) was used to measure the RNA content. The Bioanalyzer 2100 system equipped with the RNA Nano 6000 Assay Kit (Agilent Technologies, CA, USA) was adopted for assessing RNA integrity.

Three micrograms of RNA was collected from every sample and utilized as the input material to prepare RNA. First, the Epicentre Ribo-zero™ rRNA Removal Kit (Epicentre, USA) was used to remove ribosomal RNA (rRNA), while the free residue of rRNA was eliminated through ethanol precipitation. Thereafter, the NEBNext® Ultra™ Directional RNA Library Prep Kit for Illumina® (NEB, USA) was utilized to generate the sequencing libraries based on the r-RNA-free RNA, in accordance with manufacturer protocols. In brief, divalent cations were fragmented at increasing temperature using NEBNext First Strand Synthesis Reaction Buffer (5X). Thereafter, M-MuLV Reverse Transcriptase (RNaseH-) and random hexamer primers were adopted for preparing first-strand cDNA. Second-strand cDNA was synthesized by RNase H and DNA Polymerase I. dUTP was used to replace dNTPs containing dTTP in reaction buffer. The remaining overhangs were changed to blunt ends through the activities of exonuclease/polymerase. Following DNA fragment 3′-end adenylation, the NEBNext adaptor that possessed the hairpin loop structure was ligated for subsequent hybridization. To preferentially screen cDNA fragments with lengths of 150 ~ 200 bp, the AMPure XP system (Beckman Coulter, Beverly, USA) was used to purify library fragments. Thereafter, 3 μl USER Enzyme (NEB, USA) was adopted for a 15 min reaction with the cDNA selected based on size and ligated with the adaptor at 37 °C, followed by a 5 min reaction at 95 °C prior to PCR. Later, PCR was carried out using Index (X) Primer, Universal PCR primers, and Phusion High-Fidelity DNA polymerase. Finally, the Agilent Bioanalyzer 2100 system was used to purify products (AMPure XP system) and assess library quality.

The cBot Cluster Generation System was utilized to cluster those index-coded samples by the use of the TruSeq PE Cluster Kit v3-cBot-HS (Illumina) in accordance with manufacturer protocols. Following the generation of clusters, the Illumina HiSeq 4000 platform (Novogene Bioinformatics Technology Co., Ltd., Beijing, China) was used to sequence all libraries.

### Genomic characterization for lncRNAs

The raw reads in the format of fastq were first processed by the in-house Perl scripts, and clean reads were acquired through the removal of adapter-containing, poly-N-containing reads, as well as low-quality reads from the raw reads. The GC level, Q20, and Q30 in clean reads were determined. Every downstream analysis was carried out on the basis of high-quality clean data.

The annotation files of the gene model and reference genome were obtained directly from the genome websites. Bowtie2 v2.2.8 software was utilized to construct the reference genome indexes, and HISAT2 [63] v2.0.4 software was applied to align the paired-end clean data to the reference genome. The ‘--rna-strandness RF’ was set when running HISAT2, and the remaining parameters were set to be default values.

StringTie (v1.3.1) [64] software was used to assemble the mapped reads for every sample based on the reference. In StringTie, one new network flow algorithm, together with one optional de novo assembly process, is utilized for assembling and quantifying the full-length transcripts that represent several splice variants of every gene locus.

The Coding-Non-Coding-Index (CNCI) (v2) depicts the neighboring nucleotide triplets for the effective distinguishing of protein-encoding sequences from those noncoding ones that are free from those known annotations [65]. In this study, CNCI was utilized with default parameters. In addition, the Coding Potential Calculator (CPC) (0.9-r2) assesses the ORF quality and extent within one transcript and searches for sequences based on the identified protein sequence database, thus clarifying those noncoding and coding transcripts [66]. This study employed the protein database of NCBI eukaryotes, and the e-value was set as ‘1e-10’. Every transcript in all 3 potential frames was translated, and Pfam Scan (v1.3) was employed to identify the occurrence frequency for each known protein family domain from the Pfam database (release 27; both Pfam A and Pfam B were used) [67]. The transcript that had one Pfam hit was eliminated during subsequent steps. The default parameters, including -E 0.001 and --domE 0.001, were used in Pfam searches [67]. In addition, PhyloCSF (phylogenetic codon substitution frequency) (v20121028) can determine the evolutionary signatures that are specific for aligning those conserved coding regions, such as the greater synonymous codon substitution, as well as conservative amino acid substitution frequency, together with the decreased other missense or nonsense substitution frequency, thus distinguishing the protein-encoding transcripts from the noncoding ones [68]. The genome sequence alignments for multiple species were built in this study, and phyloCSF was run using the default parameters. Transcripts that were estimated by any one of those four approaches to have coding ability were eliminated, while those showing no coding ability were enrolled as candidate lncRNAs.

The Phast (v1.3) software package has covered many statistical programs, and it is frequently adopted for phylogenetic analysis [69]; in addition, phastCons has been developed as the conservation scoring and identification program for those conservative elements. In this study, phyloFit was utilized for computing the phylogenetic models for those nonconserved and conserved regions across different species and for presenting the HMM transition and model parameters to phyloP for calculating the conservation scores for various lncRNAs and protein-encoding genes.

Using Asprofile v1.0 software, alternative splicing (AS) events were clustered into 12 basic types. Thereafter, the AS event number for every sample was predicted.

### lncRNA prediction and alkaline-responsive lncRNA identification

This study adopted Cuffdiff (v2.1.1) for calculating the FPKMs (fragments per kilobase of exon model per million mapped fragments) of lncRNAs as well as coding genes for all samples [70]. Typically, the gene FPKMs were calculated by adding all transcript FPKMs for every gene group. FPKM was determined according to fragment length and number of mapped reads.

The Ballgown suite is able to interactively explore transcriptome assembly, visualize the transcript structure as well as feature-specific abundance at every locus, and annotate those assembled features into annotated ones [71]. Transcripts that had an adjusted *P*-value of < 0.05 were deemed to show differential expression. In Cuffdif, a model is used to offer statistical routines to determine the different expression patterns of digital transcripts or data on gene expression profiles according to the negative binomial distribution [70]. Transcripts that had an adjusted *P*-value of < 0.05 were deemed to show differential expression.

### Prediction of target genes

The role of Cis suggests that a lncRNA acts on adjacent target genes. In this study, the coding genes were searched 10 k/100 k upstream and downstream of lncRNAs; later, the specific functions were analyzed subsequently. By contrast, the trans role indicates that a lncRNA identifies each other based on the expression quantity. The expression correlations of lncRNAs with coding genes that had custom scripts were calculated given the small sample size of < 25; otherwise, genes obtained based on diverse samples were clustered using WGCNA [72] to identify the common expression modules; afterwards, functional enrichment analysis was carried out to examine their functions.

### Gene ontology (GO) analysis

GO analysis for those DEGs or target genes of lncRNAs was carried out using the GO seq R package after correcting the bias of gene length [73]. A GO term that had the corrected *P*-value of < 0.05 was deemed to show significant enrichment by DEGs.

### Real-time quantitative PCR (qRT-PCR)

Total RNA was extracted based on leaves from the 0-day, 3-day and 7-day alkaline-treated plants and transcribed in reverse to prepare cDNA, which was then utilized to measure the expression levels of lncRNAs responsive to alkaline stress, alkaline-responsive lncRNAs targeting miRNAs, and candidate alkaline-responsive lncRNA target genes through qRT-PCR. The ABI StepOne Plus device with the SG Fast qPCR Master Mix kit was used for qRT-PCR. Primer Express 5.0 was utilized in primer design, and primer pair specificity was determined through PCR product sequencing. Each qRT-PCR amplification was conducted three times using the uniform reaction procedure, and amplified fragment specificity was determined by generating the melting curve. Opticon Monitor Analysis Software 3.1 was applied to analyze the real-time data produced according to the 2^–ΔΔCt^ approach [74]. Additional file Table S4 shows the primers utilized in qRT-PCR.

## Supplementary information

**Additional file 1: Table S1.** LncRNAs with differential expression responding to short-term and long-term alkaline treatments.

**Additional file 2: Table S2.** Target genes of alkaline-responsive lncRNAs.

**Additional file 3: Table S3.** The components of Hoagland solutions.

**Additional file 4: Table S4.** The primers used in qRT-PCR.

**Additional file 5: Figure. S1.** Effects of alkaline treatment on growth characteristics (the scale of this photo was 1:4). (a) Plant morphology growing by means of in the absence or presence of 7 days of 75 mM alkaline treatment (b-e) Variations of transpiration rate (Tr), stomatal conductance (Gs), net photosynthetic rate (Pn), and photosystem II (Y(II)) quantum yield at 7 day after alkaline treatment. C stands for controls, while A indicates the alkaline-challenged leaves. Error bar indicates SDs across 3 biological duplicates (*n* = 3). Asterisk represents difference with statistical significance in alkaline-challenged samples compared with controls (*P < 0.05; **P < 0.01).

## Data Availability

The sequencing data have been submitted to the NCBI Gene Expression Omnibus (GEO accession number GSE107627).

## Abbreviations

lncRNAs: Long noncoding RNAs
ncRNAs: Noncoding RNAs
miRNAs: MicroRNAs
siRNAs: Small interfering RNAs
FPKM: Fragments per kilobase of exon model per million mapped fragments
qRT-PCR: Quantitative real-time PCR
ROS: Reactive oxygen species
POD: Peroxidase
GO: Gene ontology
