# Supplementary material for: Long non-coding RNAs in the alkaline stress response in sugar beet (Beta vulgaris L.)
Source: BMC Plant Biol. 2020 May 20;20:227. doi: 10.1186/s12870-020-02437-w (PMC7241001; doi:10.1186/s12870-020-02437-w)
Supplement: Supplementary file 1 — Additional file 1: Table S1. LncRNAs with differential expression responding to short-term and long-term alkaline treatments. [file 12870_2020_2437_MOESM1_ESM.docx]

Supplementary Table S1

| transcript_id | | ST_FPKM | | C_FPKM | | log2(foldchange) | | pvalue | | qvalue | |
| --- | --- | --- | --- | --- | --- | --- | --- | --- | --- | --- | --- |
| LNC_002627 | | 4.554111 | | 1.993489333 | | 1.19187357 | | 0.000229254 | | 0.251576388 | |
| LNC_007730 | | 295.987142 | | 459.5936077 | | -0.634824227 | | 0.000260161 | | 0.251576388 | |
| LNC_007229 | | 4.491383333 | | 7.710686333 | | -0.779699422 | | 0.000626909 | | 0.251576388 | |
| LNC_005253 | | 0.741869667 | | 4.563133667 | | -2.620787257 | | 0.000633642 | | 0.251576388 | |
| LNC_008123 | | 4.04103 | | 0 | | inf | | 0.000775274 | | 0.251576388 | |
| LNC_008261 | | 52.93287667 | | 84.81038933 | | -0.680076947 | | 0.001797154 | | 0.27098051 | |
| LNC_004237 | | 23.71673 | | 4.862199 | | 2.286224262 | | 0.001895867 | | 0.274569296 | |
| LNC_004675 | | 4.170634333 | | 8.475021667 | | -1.022950229 | | 0.003391656 | | 0.290488244 | |
| LNC_008076 | | 195.9285837 | | 274.988734 | | -0.489044629 | | 0.005244327 | | 0.319399245 | |
| LNC_005712 | | 160.4212647 | | 86.762044 | | 0.886729445 | | 0.006545799 | | 0.319399245 | |
| LNC_000560 | | 44.29526133 | | 27.195029 | | 0.703809405 | | 0.006758645 | | 0.319399245 | |
| LNC_008363 | | 14.17319667 | | 9.782914667 | | 0.534828922 | | 0.00681787 | | 0.319399245 | |
| LNC_006177 | | 41.631794 | | 80.36407333 | | -0.948864962 | | 0.0074815 | | 0.324572715 | |
| LNC_003697 | | 38.41820767 | | 53.24355567 | | -0.470816705 | | 0.007511738 | | 0.324572715 | |
| LNC_003819 | | 5.090626667 | | 0.231513 | | 4.458678154 | | 0.007999171 | | 0.330612399 | |
| LNC_005750 | | 3.000882 | | 0.309600333 | | 3.276907661 | | 0.008440426 | | 0.333128526 | |
| LNC_000365 | | 733.8992307 | | 1253.996094 | | -0.772878964 | | 0.009425141 | | 0.339970337 | |
| LNC_007185 | | 46.074215 | | 20.03302133 | | 1.201579564 | | 0.010047068 | | 0.343857441 | |
| LNC_005713 | | 2.923016333 | | 0.902914333 | | 1.694796871 | | 0.011297056 | | 0.350362688 | |
| LNC_008054 | | 138.203214 | | 209.8424887 | | -0.602515656 | | 0.011400655 | | 0.350362688 | |
| LNC_006920 | | 115.6238987 | | 171.0194447 | | -0.564720743 | | 0.011969849 | | 0.350362688 | |
| LNC_003352 | | 11.88440267 | | 2.139197667 | | 2.47392769 | | 0.011989613 | | 0.350362688 | |
| LNC_008260 | | 40.802503 | | 64.81960933 | | -0.66777267 | | 0.013040148 | | 0.352328036 | |
| LNC_002395 | | 523.2810263 | | 921.3698733 | | -0.816194477 | | 0.013129148 | | 0.352328036 | |
| LNC_001910 | | 12.365189 | | 4.542432333 | | 1.444747363 | | 0.014518968 | | 0.362259451 | |
| LNC_008208 | | 138.3637997 | | 213.5898237 | | -0.626376375 | | 0.015189196 | | 0.362259451 | |
| LNC_006017 | | 11.173617 | | 1.504858333 | | 2.892396691 | | 0.015775891 | | 0.362259451 | |
| LNC_003617 | | 31.30241333 | | 40.93870567 | | -0.387191601 | | 0.016184437 | | 0.362259451 | |
| LNC_008199 | | 435.8680723 | | 737.5483807 | | -0.758846159 | | 0.016837161 | | 0.362259451 | |
| LNC_005390 | | 7.815250667 | | 2.078728 | | 1.910591152 | | 0.017446428 | | 0.362259451 | |
| LNC_005395 | | 19.30313567 | | 0 | | inf | | 0.018090531 | | 0.362259451 | |
| LNC_007646 | | 5.513039 | | 3.848891667 | | 0.518404743 | | 0.018402975 | | 0.362259451 | |
| LNC_004079 | | 1955.214274 | | 3518.582275 | | -0.847667525 | | 0.019185801 | | 0.366594046 | |
| LNC_008534 | | 9.336788 | | 7.670440667 | | 0.283616863 | | 0.021185486 | | 0.370828207 | |
| LNC_000989 | | 3898.584798 | | 6997.035807 | | -0.84379336 | | 0.021599903 | | 0.370828207 | |
| LNC_006013 | | 13.074105 | | 9.003523667 | | 0.538150551 | | 0.022788381 | | 0.374934151 | |
| LNC_008464 | | 116.377327 | | 212.9882 | | -0.871963489 | | 0.023087222 | | 0.377378193 | |
| LNC_008094 | | 28.070011 | | 50.28022267 | | -0.84096141 | | 0.023408331 | | 0.377378193 | |
| LNC_007880 | | 3.194781667 | | 1.037648 | | 1.622400217 | | 0.02568505 | | 0.379861931 | |
| LNC_007400 | | 4.119113333 | | 2.123889667 | | 0.955624999 | | 0.026567754 | | 0.38140262 | |
| LNC_001677 | | 79.90322133 | | 115.7582853 | | -0.534789885 | | 0.027711188 | | 0.3826325 | |
| LNC_006119 | | 230.105138 | | 320.8189697 | | -0.479466252 | | 0.02776998 | | 0.3826325 | |
| LNC_008194 | | 1618.533569 | | 3181.776449 | | -0.975145187 | | 0.02845548 | | 0.387136848 | |
| LNC_006668 | | 27.388963 | | 30.426837 | | -0.151749724 | | 0.029558425 | | 0.390123128 | |
| LNC_005332 | | 32.88800133 | | 90.719803 | | -1.463856172 | | 0.029790881 | | 0.390123128 | |
| LNC_001194 | | 2.232284667 | | 0.55209 | | 2.01554564 | | 0.030032839 | | 0.391219275 | |
| LNC_007929 | | 15.709123 | | 22.14006933 | | -0.495057099 | | 0.03266618 | | 0.393485183 | |
| LNC_008366 | | 5.239123 | | 0.076399 | | 6.099627768 | | 0.03298492 | | 0.393485183 | |
| LNC_008093 | | 23.965374 | | 40.5894 | | -0.760151559 | | 0.03387894 | | 0.393485183 | |
| LNC_000611 | | 99.06515267 | | 134.1536843 | | -0.43743711 | | 0.034904591 | | 0.393485183 | |
| LNC_005880 | | 539.6984353 | | 884.7649333 | | -0.713140702 | | 0.036117323 | | 0.394438391 | |
| LNC_000921 | | 2.419993 | | 8.565438667 | | -1.823524259 | | 0.03613578 | | 0.394438391 | |
| LNC_004677 | | 17.24048567 | | 12.93892767 | | 0.414082359 | | 0.039123402 | | 0.399716379 | |
| LNC_008294 | | 41.87602233 | | 60.30669633 | | -0.526193793 | | 0.039172908 | | 0.399716379 | |
| LNC_000773 | | 968.353536 | | 1100.315226 | | -0.184311134 | | 0.041464198 | | 0.399716379 | |
| LNC_003245 | | 1.941032667 | | 0.257373333 | | 2.914889911 | | 0.041719912 | | 0.399716379 | |
| LNC_004949 | | 24.93044933 | | 65.611459 | | -1.396038917 | | 0.041831473 | | 0.399716379 | |
| LNC_007622 | | 75.29234067 | | 94.556712 | | -0.32867676 | | 0.041907157 | | 0.399716379 | |
| LNC_005227 | | 302.283437 | | 360.071696 | | -0.252382267 | | 0.045503449 | | 0.400609253 | |
| LNC_007731 | | 12.22378267 | | 26.68594233 | | -1.126389158 | | 0.046126025 | | 0.402986503 | |
| LNC_008048 | | 0.185011667 | | 1.904927667 | | -3.364048063 | | 0.048363366 | | 0.40804011 | |
| LNC_000443 | | 17.71749233 | | 24.27347733 | | -0.454206374 | | 0.049059969 | | 0.408177878 | |
| LNC_008060 | | 156.7735443 | | 253.454539 | | -0.693044877 | | 0.049346958 | | 0.408177878 | |
| LNC_000388 | | 284.6105347 | | 313.896169 | | -0.141298358 | | 0.049455869 | | 0.408177878 | |
| transcript_id | | LT_FPKM | | C_FPKM | | log2(foldchange) | | pvalue | | qvalue | |
| LNC_004748 | | 62.51307933 | | 0 | | inf | | 0.000258578 | | 0.790973677 | |
| LNC_003307 | | 3668.167562 | | 1832.938151 | | 1.000901437 | | 0.002164469 | | 0.99045639 | |
| LNC_008366 | | 23.945376 | | 0.076399 | | 8.29197762 | | 0.003104843 | | 0.99045639 | |
| LNC_007731 | | 11.13059467 | | 26.68594233 | | -1.261549284 | | 0.004943204 | | 0.99045639 | |
| LNC_004394 | | 1789.14624 | | 703.583598 | | 1.346477558 | | 0.005749188 | | 0.99045639 | |
| LNC_008534 | | 12.097858 | | 7.670440667 | | 0.657370264 | | 0.005765455 | | 0.99045639 | |
| LNC_006426 | | 5.930921333 | | 4.171288667 | | 0.507763081 | | 0.005776442 | | 0.99045639 | |
| LNC_008223 | | 9.357818333 | | 3.528073333 | | 1.407291673 | | 0.006061613 | | 0.99045639 | |
| LNC_004744 | | 4.355528333 | | 0 | | inf | | 0.006435973 | | 0.99045639 | |
| LNC_005725 | | 4.143576667 | | 0.009964333 | | 8.699887616 | | 0.006659244 | | 0.99045639 | |
| LNC_002468 | | 8.734804667 | | 6.807065667 | | 0.359742414 | | 0.008104041 | | 0.99045639 | |
| LNC_004676 | | 28.149948 | | 9.954623 | | 1.499693673 | | 0.009692137 | | 0.99045639 | |
| LNC_001063 | | 1364.995646 | | 1235.765381 | | 0.143491486 | | 0.010491304 | | 0.99045639 | |
| LNC_005953 | | 0 | | 2.615685 | | - | | 0.010689969 | | 0.99045639 | |
| LNC_004275 | | 26.075735 | | 2.081728333 | | 3.646854205 | | 0.011569546 | | 0.99045639 | |
| LNC_000817 | | 147.916875 | | 78.87270667 | | 0.907188593 | | 0.01265197 | | 0.99045639 | |
| LNC_004675 | | 1.072509 | | 8.475021667 | | -2.982227302 | | 0.014394147 | | 0.99045639 | |
| LNC_008363 | | 20.91632 | | 9.782914667 | | 1.096292785 | | 0.01442036 | | 0.99045639 | |
| LNC_003476 | | 3.050699 | | 6.303089333 | | -1.046919268 | | 0.016537454 | | 0.99045639 | |
| LNC_005044 | | 8.521742333 | | 0 | | inf | | 0.017122767 | | 0.99045639 | |
| LNC_000160 | | 3.561450667 | | 0 | | inf | | 0.018756233 | | 0.99045639 | |
| LNC_005944 | | 0.031392333 | | 1.139402667 | | -5.181721612 | | 0.019650442 | | 0.99045639 | |
| LNC_004747 | | 13.80237367 | | 0.085955 | | 7.327119117 | | 0.021437813 | | 0.99045639 | |
| LNC_008179 | | 7.709635 | | 5.383243 | | 0.51818701 | | 0.025493877 | | 0.99045639 | |
| LNC_000301 | | 37.06782867 | | 19.75908 | | 0.907651833 | | 0.035139445 | | 0.99045639 | |
| LNC_001109 | | 26.05185633 | | 18.59781433 | | 0.486253094 | | 0.035288116 | | 0.99045639 | |
| LNC_002429 | | 135.4185637 | | 91.53285233 | | 0.565063979 | | 0.035700609 | | 0.99045639 | |
| LNC_007400 | | 5.855586667 | | 2.123889667 | | 1.463104899 | | 0.036295514 | | 0.99045639 | |
| LNC_001019 | | 32.40387233 | | 39.249949 | | -0.276524551 | | 0.036501743 | | 0.99045639 | |
| LNC_008411 | | 13.77495267 | | 16.99704833 | | -0.303236872 | | 0.037986693 | | 0.99045639 | |
| LNC_008308 | | 7.478338667 | | 8.200014667 | | -0.132908683 | | 0.039477366 | | 0.99045639 | |
| LNC_008369 | | 0 | | 2.999022 | | - | | 0.040420961 | | 0.99045639 | |
| LNC_001194 | | 4.334338667 | | 0.55209 | | 2.972836509 | | 0.041035229 | | 0.99045639 | |
| LNC_007000 | | 13.48057033 | | 12.86317233 | | 0.067635049 | | 0.043929858 | | 0.99045639 | |
| LNC_000365 | | 512.3509117 | | 1253.996094 | | -1.291328691 | | 0.044063634 | | 0.99045639 | |
| LNC_000401 | | 4.072077333 | | 1.058974667 | | 1.943096883 | | 0.04473659 | | 0.99045639 | |
| LNC_004841 | | 0 | | 3.549599667 | | - | | 0.046163571 | | 0.99045639 | |
