# Supplementary material for: Long non-coding RNAs in the alkaline stress response in sugar beet (Beta vulgaris L.)
Source: BMC Plant Biol. 2020 May 20;20:227. doi: 10.1186/s12870-020-02437-w (PMC7241001; doi:10.1186/s12870-020-02437-w)
Supplement: Supplementary file 2 — Additional file 2: Table S2. Target genes of alkaline-responsive lncRNAs. [file 12870_2020_2437_MOESM2_ESM.docx]

Supplementary Table S2

| lncRNA ID | lncRNA Strand | Target Gene Symbol | Target Gene Prediction |
| --- | --- | --- | --- |
| LNC_000160 | + | LOC104893173 | protein translocase subunit SECA2 |
| LNC_000160 | + | LOC104893195 | NAD kinase 2 |
| LNC_000301 | - | LOC104882882 | cyclic pyranopterin monophosphate synthase accessory protein |
| LNC_000301 | - | LOC104883533 | calcineurin B-like protein 4 |
| LNC_000365 | - | LOC104891044 | kinesin-like protein KIN-7E |
| LNC_000365 | - | LOC104891062 | zinc finger BED domain-containing protein RICESLEEPER 2 |
| LNC_000365 | - | LOC104891096 | glutamic acid-rich protein |
| LNC_000365 | - | LOC104891044 | kinesin-like protein KIN-7E |
| LNC_000401 | - | LOC104892827 | glycerophosphodiester phosphodiesterase GDPD4 |
| LNC_000401 | - | LOC104892804 | centrosomal protein of 128 kDa |
| LNC_000401 | - | LOC104892844 | RING-H2 finger protein ATL7 |
| LNC_000401 | - | LOC104892857 | hypothetical protein isoform B |
| LNC_000401 | - | LOC104892793 | ferredoxin-thioredoxin reductase |
| LNC_000401 | - | LOC104892868 | single-stranded DNA-bindig protein WHY2 |
| LNC_000443 | - | LOC104897950 | agamous-like MADS-box protein AGL12 |
| LNC_000560 | + | LOC104906857 | receptor-like protein kinase FERONIA |
| LNC_000560 | + | LOC104906921 | protein COBRA-like |
| LNC_000611 | + | LOC104883280 | neurofilament heavy polypeptide |
| LNC_000611 | + | LOC104883214 | chaperone protein ClpB4 |
| LNC_000611 | + | LOC104883248 | hypothetical protein isoform B |
| LNC_000773 | + | LOC104887294 | potassium channel SKOR-like |
| LNC_000773 | + | LOC104887297 | chorismate mutase 3 |
| LNC_000817 | + | LOC104887656 | pentatricopeptide repeat-containing protein At5g39980 |
| LNC_000817 | + | LOC104887653 | pentatricopeptide repeat-containing protein At1g59720 |
| LNC_000817 | + | LOC104887645 | superoxide dismutase [Mn] |
| LNC_000921 | - | LOC104885095 | chromatin modification-related protein eaf-1 |
| LNC_000921 | - | LOC104885066 | pentatricopeptide repeat-containing protein At5g13770 |
| LNC_000921 | - | LOC104884878 | exosome complex component RRP45B |
| LNC_000921 | - | LOC104885084 | D-amino-acid transaminase |
| LNC_000921 | - | LOC104884912 | myb-related protein 3R-1 |
| LNC_001109 | - | LOC104887428 | ras-related protein RABF1 |
| LNC_001109 | - | LOC104887425 | AAA-ATPase At3g28510 |
| LNC_001194 | + | LOC104888232 | hypothetical protein isoform B |
| LNC_001194 | + | LOC104888226 | myosin-2 heavy chain |
| LNC_001194 | + | LOC104888235 | starch synthase 1 |
| LNC_001194 | + | LOC104888216 | KH domain-containing protein At4g18375 |
| LNC_001194 | + | LOC104888218 | kinesin light chain |
| LNC_001194 | + | LOC104888217 | protein MITOFERRINLIKE 1 |
| LNC_001194 | + | LOC104888228 | protein ABIL3 |
| LNC_001194 | + | LOC104888230 | ferrochelatase-2 |
| LNC_001677 | + | LOC104890608 | meiotic recombination protein DMC1 homolog |
| LNC_001910 | - | LOC104890333 | protein FAM133 |
| LNC_001910 | - | LOC104890340 | hypothetical protein isoform A |
| LNC_002429 | + | LOC104894028 | thioredoxin-like 1-2 |
| LNC_002429 | + | LOC104894029 | histidine biosynthesis bifunctional protein hisIE |
| LNC_002429 | + | LOC104894033 | agamous-like MADS-box protein AGL65 |
| LNC_002429 | + | LOC104894032 | chlorophyll a-b binding protein 4 |
| LNC_002468 | + | LOC104894297 | diphthine methyltransferase homolog |
| LNC_002468 | + | LOC104894300 | protein MICRORCHIDIA 6 |
| LNC_002468 | + | LOC104894293 | anthranilate phosphoribosyltransferase |
| LNC_002468 | + | LOC104894410 | tRNA(His) guanylyltransferase 1-like |
| LNC_002627 | - | LOC104892833 | F-box protein CPR30 |
| LNC_002627 | - | LOC104892834 | DNA repair endonuclease UVH1 |
| LNC_002627 | - | LOC104892835 | glucan endo-1 |
| LNC_002627 | - | LOC104892832 | cationic amino acid transporter 7 |
| LNC_003245 | + | LOC104897206 | cyclin-D-binding Myb-like transcription factor 1 |
| LNC_003307 | + | LOC104897586 | mannosyl-oligosaccharide glucosidase GCS1 |
| LNC_003307 | + | LOC104897576 | flavonoid 3' |
| LNC_003307 | + | LOC104897579 | endoplasmic reticulum metallopeptidase 1 |
| LNC_003307 | + | LOC104897575 | ELMO domain-containing protein B |
| LNC_003307 | + | LOC104897567 | CTD small phosphatase-like protein 2 |
| LNC_003307 | + | LOC104897584 | F-box/kelch-repeat protein At1g67480 |
| LNC_003352 | + | LOC104897949 | homeobox-leucine zipper protein ATHB-13 |
| LNC_003352 | + | LOC104897956 | UDP-galactose/UDP-glucose transporter 3 |
| LNC_003352 | + | LOC104897953 | hypothetical protein |
| LNC_003352 | + | LOC104897957 | protein kinase 2B |
| LNC_003476 | - | LOC104895762 | squamosa promoter-binding-like protein 6 |
| LNC_003476 | - | LOC104895763 | protein CRABS CLAW |
| LNC_003476 | - | LOC104895769 | folate synthesis bifunctional protein |
| LNC_003476 | - | LOC104895760 | 15-cis-phytoene desaturase |
| LNC_003476 | - | LOC104895770 | fructokinase-like 2 |
| LNC_003697 | - | LOC104896979 | cytokinin riboside 5'-monophosphate phosphoribohydrolase LOG3 |
| LNC_003697 | - | LOC104896980 | probable leucine-rich repeat receptor-like protein kinase At5g49770 |
| LNC_003819 | - | LOC104897608 | protein UXT homolog |
| LNC_003819 | - | LOC104897610 | NADH dehydrogenase [ubiquinone] flavoprotein 1 |
| LNC_003819 | - | LOC104897612 | probable pre-mRNA-splicing factor ATP-dependent RNA helicase DEAH4 |
| LNC_003819 | - | LOC104897606 | protein UXT homolog |
| LNC_004237 | - | LOC104898212 | alanine--glyoxylate aminotransferase 2 homolog 3 |
| LNC_004237 | - | LOC104898220 | pentatricopeptide repeat-containing protein At2g38420 |
| LNC_004237 | - | LOC104898219 | lysM domain receptor-like kinase 3 |
| LNC_004394 | - | LOC104899331 | 39S ribosomal protein L47 |
| LNC_004675 | + | LOC104901469 | BTB/POZ domain-containing protein NPY2 |
| LNC_004675 | + | LOC104901533 | myosin heavy chain |
| LNC_004676 | + | LOC104901469 | BTB/POZ domain-containing protein NPY2 |
| LNC_004676 | + | LOC104901533 | myosin heavy chain |
| LNC_004677 | + | LOC104901469 | BTB/POZ domain-containing protein NPY2 |
| LNC_004677 | + | LOC104901533 | myosin heavy chain |
| LNC_004744 | + | LOC104902121 | protein argonaute PNH1 |
| LNC_004744 | + | LOC104902115 | 3-ketoacyl-CoA synthase 11 |
| LNC_004744 | + | LOC104902111 | EG45-like domain containing protein |
| LNC_004744 | + | LOC104902110 | uncharacterized LOC104902110 |
| LNC_004747 | + | LOC104902122 | RNA-binding protein 25 |
| LNC_004747 | + | LOC104902121 | protein argonaute PNH1 |
| LNC_004747 | + | LOC104902115 | 3-ketoacyl-CoA synthase 11 |
| LNC_004748 | + | LOC104902122 | RNA-binding protein 25 |
| LNC_004748 | + | LOC104902121 | protein argonaute PNH1 |
| LNC_004748 | + | LOC104902115 | 3-ketoacyl-CoA synthase 11 |
| LNC_004841 | - | LOC104900832 | putative yippee-like protein Os10g0369500 |
| LNC_004841 | - | LOC104900873 | inositol transporter 4 |
| LNC_004949 | - | LOC104901422 | hypothetical protein isoform B |
| LNC_004949 | - | LOC104901423 | alcohol dehydrogenase-like 6 |
| LNC_004949 | - | LOC104901424 | DNA ligase 1 |
| LNC_005044 | - | LOC104902121 | protein argonaute PNH1 |
| LNC_005044 | - | LOC104902115 | 3-ketoacyl-CoA synthase 11 |
| LNC_005044 | - | LOC104902111 | EG45-like domain containing protein |
| LNC_005332 | + | LOC104904044 | chaperone protein dnaJ A6 |
| LNC_005332 | + | LOC104904050 | polyadenylate-binding protein 2-like |
| LNC_005390 | + | LOC104904476 | presequence protease 2 |
| LNC_005390 | + | LOC104904478 | zinc-binding alcohol dehydrogenase domain-containing protein 2 |
| LNC_005395 | + | LOC104904478 | zinc-binding alcohol dehydrogenase domain-containing protein 2 |
| LNC_005395 | + | LOC104904476 | presequence protease 2 |
| LNC_005712 | - | LOC104904432 | pentatricopeptide repeat-containing protein At2g15630 |
| LNC_005712 | - | LOC104904427 | proteasome assembly chaperone 2 |
| LNC_005712 | - | LOC104905016 | pentatricopeptide repeat-containing protein At1g06140 |
| LNC_005713 | - | LOC104904427 | proteasome assembly chaperone 2 |
| LNC_005713 | - | LOC104904432 | pentatricopeptide repeat-containing protein At2g15630 |
| LNC_005713 | - | LOC104904441 | TATA-box-binding protein 2 |
| LNC_005713 | - | LOC104905016 | pentatricopeptide repeat-containing protein At1g06140 |
| LNC_005725 | - | LOC104904534 | protein FAR1-RELATED SEQUENCE 6 |
| LNC_005725 | - | LOC104904535 | protein FAR1-RELATED SEQUENCE 8 |
| LNC_005750 | - | LOC104904694 | serpin-Z2B |
| LNC_005750 | - | LOC104904691 | protein CURVATURE THYLAKOID 1C |
| LNC_005750 | - | LOC104904700 | probable plastidic glucose transporter 3 |
| LNC_005750 | - | LOC104904704 | calcineurin subunit B |
| LNC_005750 | - | LOC104904709 | hypothetical protein isoform B |
| LNC_005750 | - | LOC104904697 | magnesium transporter MRS2-4 |
| LNC_005880 | - | LOC104905246 | nucleolin |
| LNC_005880 | - | LOC104905248 | hypothetical protein isoform B |
| LNC_005880 | - | LOC104905245 | cytochrome c oxidase-assembly factor COX23 |
| LNC_005880 | - | LOC104905242 | putative casein kinase II subunit beta-4 |
| LNC_005944 | + | LOC104905664 | pentatricopeptide repeat-containing protein At5g42310 |
| LNC_005944 | + | LOC104905658 | metal tolerance protein 10 |
| LNC_005944 | + | LOC104905663 | glucan endo-1 |
| LNC_005944 | + | LOC104905660 | probable acyl-activating enzyme 1 |
| LNC_005944 | + | LOC104905661 | carboxypeptidase A6 |
| LNC_005953 | - | LOC104905664 | pentatricopeptide repeat-containing protein At5g42310 |
| LNC_005953 | - | LOC104905658 | metal tolerance protein 10 |
| LNC_005953 | - | LOC104905663 | glucan endo-1 |
| LNC_005953 | - | LOC104905660 | probable acyl-activating enzyme 1 |
| LNC_005953 | - | LOC104905661 | carboxypeptidase A6 |
| LNC_006013 | - | LOC104905819 | probable nucleoredoxin 2 |
| LNC_006017 | - | LOC104905826 | translation initiation factor IF-2 |
| LNC_006017 | - | LOC104905825 | hypothetical protein isoform A |
| LNC_006119 | - | LOC104906167 | nipped-B-like protein B |
| LNC_006119 | - | LOC104906164 | alpha-1 |
| LNC_006426 | + | LOC104907291 | ATP-dependent RNA helicase-like protein DB10 |
| LNC_006920 | + | LOC104908930 | hypothetical protein isoform B |
| LNC_007185 | - | LOC104883386 | DNA mismatch repair protein MSH5 |
| LNC_007185 | - | LOC104883389 | GDSL esterase/lipase At5g55050-like |
| LNC_007185 | - | LOC104883380 | WRKY transcription factor 42-like |
| LNC_007229 | - | LOC104883552 | E3 ubiquitin-protein ligase MIEL1 |
| LNC_007229 | - | LOC104883550 | CASP-like protein 1E1 |
| LNC_007229 | - | LOC104883551 | hypothetical protein isoform A |
| LNC_007229 | - | LOC104883555 | probable feruloyl esterase A |
| LNC_007400 | - | LOC104884065 | alpha-1 |
| LNC_007400 | - | LOC104884067 | cell division cycle and apoptosis regulator protein 1 |
