# Supplementary material for: Long non-coding RNAs in the alkaline stress response in sugar beet (Beta vulgaris L.)
Source: BMC Plant Biol. 2020 May 20;20:227. doi: 10.1186/s12870-020-02437-w (PMC7241001; doi:10.1186/s12870-020-02437-w)
Supplement: Supplementary file 3 — Additional file 3: Table S3. The components of Hoagland solutions. [file 12870_2020_2437_MOESM3_ESM.docx]

Supplementary Table S3

| Molecular formula | mmol/L |  | mg/L |
| --- | --- | --- | --- |
| EDTA·FeNa | 0.02 |  | 8.422 |
| MnSO_4_·H_2_O | 0.006722 |  | 1.136085 |
| CuSO_4_·5H_2_O | 0.000316 |  | 0.078899 |
| ZnSO_4_·7H_2_O | 0.000765 |  | 0.219968 |
| H_3_BO_3_ | 0.04625 |  | 2.859638 |
| H_2_MoO_4_ | 0.0005 |  | 0.089985 |
| Ca(NO_3_)_2_·4H_2_0 | 5 |  | 1180.75 |
| KNO_3_ | 5 |  | 505.55 |
| MgSO_4_·7H_2_O | 2.5 |  | 616.175 |
| KH_2_PO_4_ | 2 |  | 272.18 |
| NaH_2_PO_4_ | 2 |  | 239.96 |
| NaNO_3_ | 5 |  | 425.05 |
| MgCl_2_ | 2.5 |  | 238.025 |
| Na_2_SO_4_ | 2.5 |  | 355.1 |
| CaCl_2_ | 5 |  | 554.9 |
| KCl | 5 |  | 372.75 |
