# Supplementary material for: Long non-coding RNAs in the alkaline stress response in sugar beet (Beta vulgaris L.)
Source: BMC Plant Biol. 2020 May 20;20:227. doi: 10.1186/s12870-020-02437-w (PMC7241001; doi:10.1186/s12870-020-02437-w)
Supplement: Supplementary file 4 — Additional file 4: Table S4. The primers used in qRT-PCR. [file 12870_2020_2437_MOESM4_ESM.docx]

Supplementary Table S4

| **Actin primers** |
| --- |
| Actin-F 5' AATGTTCCCTGGTATTGCTGAC 3' 58.4 |
| Actin-R 5' CACTTTCTGTGGACGATTGATG 3' 58.0 210bp |
| **Alkaline-responsive lncRNAs primers** |
| LNC_001194-F 5' AGTTCAGGGGCAATCAATCTAT 3' 57.4 |
| LNC_001194-R 5' TTCCCGAAGGATGGATGAT 3' 57.2 137bp |
| LNC_007400-F 5' AATCACGCTGGTTGGCTGT 3' 58.5 |
| LNC_007400-R 5' ATCAAGCCAGCAACGAAAAC 3' 57.9 104bp |
| LNC_008363-F 5' GGCAGCATACTCAATCACTCG 3' 58.2 |
| LNC_008363-R 5' TGTAGCAATTCGGGTTATGGA 3' 58.3 94bp |
| LNC_008366-F 5' TCACAGTTGTAGAGTTGAATCTCGT 3' 57.9 |
| LNC_008366-R 5' AGTTGAACAGAACGCACCCT 3' 57.0 136bp |
| LNC_008534-F 5' CCTCTGGCATTACGACCTTT 3' 57.0 |
| LNC_008534-R 5' GCAATGGAGCCGTAGACAAT 3' 57.6 94bp |
| LNC_000365-F 5' TTCACTCCATAGTCTGATAGATCGT 3' 57.1 |
| LNC_000365-R 5' AATGGGACTCTATCTTTATTCTCGT 3' 57.5 70bp |
| LNC_004675-F 5' TTCCTTTCGATGAATACACGG 3' 57.9 |
| LNC_004675-R 5' AGGGAGCCATTCCTTTGACT 3' 57.8 148bp |
| LNC_007731-F 5' GAACGAATGGACAAGGAGTGA 3' 57.3 |
| LNC_007731-R 5' AAAGAGTTCGGACAATTGGGT 3' 57.9 141bp |
| **Primers of partial potential gene of alkaline-responsive lncRNAs** |
| LOC1048882226-F 5’ TTCATGACCAAGCAACTCGG 3’ 58.5 |
| LOC1048882226-R 5’ GATGCCGCTTCTCAATAGCA 3’ 58.4 81bp |
| LOC104891570-F 5' CCAAGGCTAGGGCACTCAA 3' 58.5 |
| LOC1048915570-R 5' TTCACAAGGCTTACCGTTCTC 3' 57.2 170bp |
| LOC104894889-F 5' GTACCGTAAACTCCGTCGCAT 3' 59.4 |
| LOC104894889-R 5' CCTTCGCAAATCCAAACACTT 3' 59.6 162bp |
| LOC104903711-F 5' AGTGCCCTGATGATGTATATGTCT 3' 57.5 |
| LOC104903711-R 5' AGCACAAGTAAGAACCCATCCT 3' 57.5 185bp |
