# Supplementary material for: Long non-coding RNAs in the alkaline stress response in sugar beet (Beta vulgaris L.)
Source: BMC Plant Biol. 2020 May 20;20:227. doi: 10.1186/s12870-020-02437-w (PMC7241001; doi:10.1186/s12870-020-02437-w)
Supplement: Supplementary file 5 — Additional file 5: Figure. S1. Effects of alkaline treatment on growth characteristics (the scale of this photo was 1:4). (a) Plant morphology growing by means of in the absence or presence of 7 days of 75 mM alkaline treatment (b-e) Variations of transpiration rate (Tr), stomatal conductance (Gs), net photosynthetic rate (Pn), and photosystem II (Y(II)) quantum yield at 7 day after alkaline treatment. C stands for controls, while A indicates the alkaline-challenged leaves. Error bar indicates SDs across 3 biological duplicates (n = 3). Asterisk represents difference with statistical significance in alkaline-challenged samples compared with controls (*P < 0.05; **P < 0.01). [file 12870_2020_2437_MOESM5_ESM.docx]

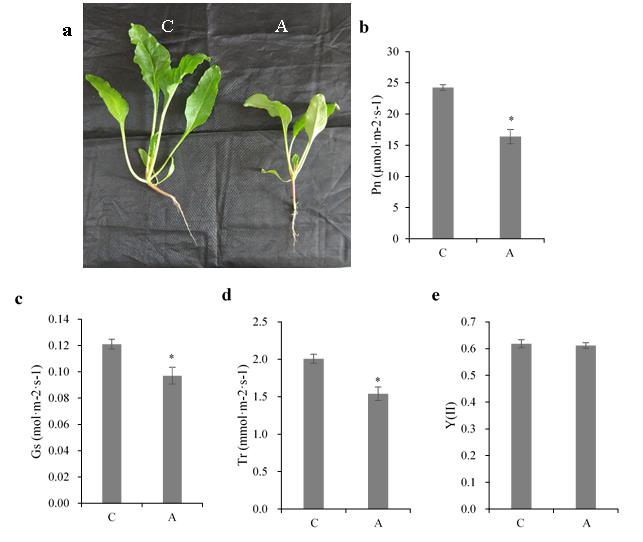


**Fig. S1.** Effects of alkaline treatment on growth characteristics (the scale of this photo was 1:4). (a) Plant morphology growing by means of in the absence or presence of 7 days of 75 mM alkaline treatment (b-e) Variations of transpiration rate (Tr) , stomatal conductance (Gs), net photosynthetic rate (Pn), and photosystem II (Y(II)) quantum yield at 7 day after alkaline treatment. C stands for controls, while A indicates the alkaline-challenged leaves. Error bar indicates SDs across 3 biological duplicates (n=3). Asterisk represents difference with statistical significance in alkaline-challenged samples compared with controls (*P<0.05; **P<0.01).
